# An Improved RODNet for Object Detection Based on Radar and Camera Fusion

**DOI:** 10.3390/s26020373

**Published:** 2026-01-06

**Authors:** Manman Fan, Xianpeng Wang, Mingcheng Fu, Yanqiu Yang, Yuehao Guo, Xiang Lan

**Affiliations:** School of Information and Communication Engineering, Hainan University, Haikou 570228, China; 23210810000010@hainanu.edu.cn (M.F.); fumcheng@hainanu.edu.cn (M.F.); 25120854020004@hainanu.edu.cn (Y.Y.); gyhao2024@hainanu.edu.cn (Y.G.); xlan@hainanu.edu.cn (X.L.)

**Keywords:** sensor fusion, millimeter-wave radar, radar–camera fusion, sensor calibration, cross-device generalization, temporal attention, object detection, autonomous driving

## Abstract

Deep learning-based radar detection often suffers from poor cross-device generalization due to hardware heterogeneity. To address this, we propose a unified framework that combines rigorous calibration with adaptive temporal modeling. The method integrates three coordinated steps: (1) ensuring precise spatial alignment via improved Perspective-n-Point (PnP) calibration with closed-loop verification; (2) unifying signal statistics through multi-range bin calibration and chirp-wise Z-score standardization; and (3) enhancing feature consistency using a lightweight global–temporal adapter (GTA) driven by global gating and three-point attention. By combining signal-level standardization with feature-level adaptation, our framework achieves 86.32% average precision (AP) on the ROD2021 dataset. It outperforms the E-RODNet baseline by 22.88 percentage points with a 0.96% parameter increase, showing strong generalization across diverse radar platforms.

## 1. Introduction

Millimeter-wave radar sensors have become essential components in autonomous driving sensor suites due to their all-weather robustness, direct range–velocity–angle measurement capabilities, and cost-effectiveness. Unlike camera sensors, which degrade in low-light or high-glare conditions, and LiDAR sensors, constrained by high costs, mechanical complexity, and weather sensitivity [1], millimeter-wave radar sensors maintain stable sensing performance under adverse environmental conditions, including rain, fog, backlighting, and nighttime operation. These intrinsic sensor advantages have driven extensive research on radar-based perception systems for robust autonomous vehicle sensing [2,3,4].

However, radar sensor signals inherently exhibit low spatial resolutions and lack semantic features such as shape or texture, fundamentally limiting the object discrimination capabilities. Due to these sensor-level signal characteristics, traditional radar signal processing methods such as the constant false alarm rate (CFAR) [5] and clustering-based algorithms [6], which rely on classical signal processing and statistical modeling, often produce high false alarm rates in cluttered environments and lack semantic representation capabilities, making them insufficient for high-level perception tasks despite their computational efficiency. To overcome these fundamental sensor limitations, multi-sensor fusion combining radar and camera has emerged as a promising approach. By exploiting the complementary sensing modalities—radar’s robust range–velocity measurement and camera’s rich semantic information—fusion frameworks enhance perception performance through cross-modal learning while maintaining all-weather operation. Vision-guided supervision and deep learning-based sensor fusion have become effective strategies, enabling radar sensing networks to learn semantic-aware representations from synchronized camera sensor data [2,7,8,9,10]. However, cross-modal supervision alone cannot address the fundamental challenges of radar sensor signal quality variations and cross-device hardware heterogeneity.

Recent advances in deep learning have transformed radar perception from rule-based processing to data-driven paradigms. The Radar Object Detection Network (RODNet) [2] pioneered radar object detection directly from range–azimuth (RA) maps under camera supervision, providing pixel-level cross-modal training signals. E-RODNet [11] further improved spatiotemporal modeling through short-sequence fusion (SFF) and enhanced encoder–decoder architectures. Subsequent studies have explored radar–camera fusion and bird’s-eye-view (BEV) perception frameworks [9,12,13,14,15], leveraging cross-modal supervision and attention mechanisms to enrich semantic understanding. Nevertheless, despite these advances, deep learning-based radar detection still suffers from two critical bottlenecks that hinder practical deployment: (1) poor cross-device generalization due to hardware-dependent signal distributions and (2) unstable temporal consistency in dynamic driving scenes.

The first issue—cross-device domain shift—occurs because radar devices differ in antenna array configuration, frequency modulation, and noise statistics. Models trained on one sensor may perform poorly on another [16]. Although standardization-based domain adaptation techniques [17] have shown promise, existing radar frameworks still lack systematic calibration and distribution alignment mechanisms. The second issue—insufficient temporal modeling—arises because most current radar networks use local 3D convolutions with limited receptive fields and no explicit temporal attention, leading to detection jitter and inconsistency across frames [18].

To address these challenges, this paper proposes an improved RODNet-based radar–camera fusion framework for robust object detection. The framework synergistically integrates geometric alignment, time-division multiplexing multiple-input multiple-output (TDM-MIMO) channel calibration, chirp-wise Z-score standardization, and a lightweight GTA to tackle cross-device domain shift and temporal instability. These components work collaboratively: geometric alignment enables effective training supervision, calibration and standardization decouple hardware-specific characteristics from semantic features, and the GTA module stabilizes temporal predictions, collectively achieving robust cross-device object detection.

Experiments on the public ROD2021 dataset demonstrate that the proposed fusion framework achieves average precision of 86.32%, outperforming the baseline E-RODNet by 22.88 percentage points with only a 0.96% parameter increase. Experimental validation further demonstrates that channel calibration reduces the main lobe width by 42.3%, statistical standardization stabilizes the complex signal standard deviation to 0.037 ± 0.005, and the GTA module improves the detection performance with a minimal parameter overhead.

The main contributions of this paper are summarized as follows:

1. A radar–camera fusion framework with improved PnP-based extrinsic calibration and closed-loop geometric verification, enabling precise cross-modal coordinate alignment for effective training supervision.
2. A multi-range-bin joint TDM-MIMO channel calibration method that corrects receive-channel complex gains and transmit-phase deviations, improving cross-device array consistency.
3. A chirp-wise Z-score standardization strategy with formal mathematical definitions that achieves the statistical alignment of radar signal distributions (standard deviation converging to 0.037, 99th percentile at 0.19) across sensors, enhancing model generalization and transferability.
4. A lightweight GTA module that stabilizes short-term temporal features through global gating and temporal attention with only approximately 0.12 M parameters.
5. Comprehensive experiments on the ROD2021 dataset and signal-level cross-device verification on AWR1642 data, validating the effectiveness of the proposed preprocessing pipeline.

The remainder of this paper is structured as follows: Section 2 reviews related work in radar preprocessing, object detection, and attention mechanisms; Section 3 introduces the radar platform and signal modeling; Section 4 presents the proposed methodology, including geometric alignment, channel calibration, statistical standardization, and the GTA module; and Section 5 provides the experimental results and analysis. Finally, Section 6 summarizes the conclusions.

## 2. Related Work

### 2.1. Radar Data Preprocessing

Millimeter-wave radar signal processing typically involves multiple stages to transform raw time-domain signals into multi-dimensional representations. Range fast Fourier transform (FFT) extracts distance information, Doppler FFT captures velocity characteristics, and angle FFT estimates angular positions, providing essential features for object detection and tracking. The phase consistency of TDM-MIMO virtual arrays is crucial for accurate angle estimation [19]. However, different radar hardware exhibits significant variations in terms of transmit–receive channel numbers, sampling rate, bandwidth, and antenna geometry. Moreover, the amplitude distribution of raw signals is affected by environmental clutter, electromagnetic interference, and thermal noise. These factors lead to inconsistent signal characteristic distributions across device domains, significantly limiting the generalization performance of deep learning models.

Early preprocessing research mainly focused on single-device channel calibration and noise suppression. For example, correcting transmitter and receiver complex gains allows compensation for channel amplitude drift [20], while phase difference estimation-based calibration methods can alleviate the impacts of array errors on angle spectra [21]. Recent work has explored the statistical standardization of radar signals, including amplitude normalization, power spectrum compression, and adaptive noise modeling, to improve model robustness across different scenarios. However, these methods often rely on device-specific parameters or fixed environment settings and lack a unified cross-device standardization strategy. In addition, existing approaches generally ignore temporal feature statistical drift, such as chirp-level amplitude fluctuations and inter-frame power inconsistencies, which significantly weaken model performance in multi-device training and transfer scenarios.

To address these challenges, this paper proposes a general preprocessing pipeline with multi-range-bin joint channel calibration and chirp-wise Z-score standardization. The former achieves consistent array responses via the joint optimization of complex gains and phase deviations; the latter aligns cross-frame and cross-device statistical distributions. This approach improves the angle spectrum clarity and feature stability with low computational complexity.

### 2.2. Radar Object Detection

Millimeter-wave radar object detection methods include traditional signal processing algorithms and deep learning approaches. Traditional methods such as CFAR [5] and clustering [6] have high computational efficiency but struggle with clutter, occlusion, and semantic feature extraction in complex scenes.

Deep learning has transformed radar perception towards data-driven approaches. RODNet [2] pioneered learning from range–azimuth (RA) maps using 3D convolutional neural networks (CNNs) with camera pseudo-labels for cross-modal supervision. Subsequent variants (RODNet-HG, RODNet-CDC) introduced HourGlass and deformable convolution. E-RODNet [11] achieved significant improvements through short-sequence fusion (SFF) and an enhanced encoder–decoder architecture, providing a lightweight baseline. These networks leverage residual connections [22] and encoder–decoder architectures [23] for multi-scale feature fusion. T-RODNet [24] achieved 83.83% AP using Swin Transformer but requires 159.7M parameters, limiting real-time deployment.

Recent work has explored hybrid Transformer–CNN architectures. TC-Radar [25] integrates Transformers for long-range dependencies with CNNs for local features, TransRadar [26] proposes adaptive directional Transformers for multi-view segmentation, and SMIFormer [27] leverages multi-view Transformers for 4D radar detection. The K-Radar dataset [28] provides comprehensive 4D radar data for weather-robust evaluation. Recent 4D radar fusion work [29,30] combines LiDAR and radar for improved robustness in adverse conditions.

Beyond RA maps, radar point cloud methods leverage 3D architectures like PointNet++ [31], VoxelNet, and PointPillars. However, radar point clouds suffer from extreme sparsity (10–50 points/frame vs. LiDAR’s 10,000+), fundamentally limiting their effectiveness. RAMP-CNN [32] combines RA maps and point clouds for improved accuracy but at the cost of increased complexity. Recent efficient architectures include YOLOv8 [33], RT-DETR [34], and EfficientDet [35].

While existing research primarily focuses on network architecture innovation (HourGlass, Deformable Convolution, Transformers) to improve the detection performance on single-device datasets, these methods lack the systematic treatment of two fundamental challenges: (1) cross-device domain shift—existing domain adaptation methods [16,17] address high-level feature distribution alignment but ignore device-specific signal-level heterogeneity in radar systems (antenna configuration, frequency modulation, noise statistics); (2) temporal consistency—while hybrid Transformer–CNN models [25,26,27] employ global self-attention for temporal modeling, their $O(N^{2})$ complexity and heavy parameter requirements (e.g., T-RODNet with 159.7 M parameters) limit real-time deployment.

This paper addresses both challenges through a dual-level approach. At the signal level, we propose multi-range-bin joint TDM-MIMO calibration and chirp-wise Z-score standardization to achieve hardware-agnostic preprocessing that decouples device-specific characteristics from semantic features, enabling cross-device model transferability. At the feature level, we introduce a lightweight GTA module (0.12 M parameters) that achieves efficient temporal modeling through three-point attention with linear O(N) complexity, balancing performance and deployability. Unlike existing works that treat preprocessing and architectures separately, our framework synergistically integrates signal standardization and temporal attention, achieving 86.32% AP with only a 0.96% parameter increase—demonstrating that systematic signal-level preprocessing can achieve comparable or superior performance to heavy Transformer-based models while maintaining computational efficiency.

### 2.3. Attention Mechanisms

Attention mechanisms have been widely applied in computer vision and temporal modeling tasks. Vision Transformer (ViT) [36] pioneered the application of pure Transformer architectures to image recognition, demonstrating the effectiveness of self-attention for visual feature learning. The Squeeze-and-Excitation Network (SENet) [37] learns channel attention weights through global average pooling and two-layer fully connected networks, adaptively recalibrating channel-wise feature responses. The convolutional block attention module (CBAM) [38] further extends this concept by fusing both channel and spatial attention mechanisms. In temporal modeling, the temporal shift module (TSM) [39] captures temporal dependencies through efficient channel shift operations, and the temporal convolutional network (TCN) [40] uses causal convolution to construct a long-term temporal information flow. Transformer [41], based on a self-attention mechanism, can explicitly model global dependencies, but its computational complexity is $O(N^{2})$, which becomes prohibitive for high-resolution radar data.

In recent years, researchers have proposed various lightweight temporal attention structures to reduce the temporal modeling complexity while maintaining effectiveness. Lightweight Vision Transformers [42] achieve competitive performance with significantly reduced parameters through bidirectional interaction between local and global features. Drawing on the temporal neighborhood concept from TSM, this paper designs a GTA module consisting of three-point temporal attention based on roll operations and a global gating mechanism. This module explicitly models inter-frame dependencies in the temporal dimension while maintaining linear complexity O(N), achieving efficient temporal alignment and dynamic feature aggregation and significantly improving the stability and robustness of object detection in dynamic scenes.

## 3. Radar Platform and Signal Modeling

This section introduces the AWR1642 radar hardware platform and the single-chirp RA map construction process, which serve as the foundation for subsequent preprocessing and deep learning-based object detection.

### 3.1. AWR1642 Hardware Configuration and Resolution Characteristics

The TI AWR1642 operates in the 77 GHz frequency band with a two-transmit and four-receive antenna configuration, where $T_{1}$ and $T_{2}$ denote the two transmit antennas, and $R_{1},R_{2},R_{3},R_{4}$ denote the four receive antennas, forming an eight-channel virtual array through TDM-MIMO. Key configuration parameters are shown in Table 1.

The AWR1642 radar operates within the 76–81 GHz frequency band as specified in the hardware manual. The sweep bandwidth is determined by the frequency slope and chirp duration as follows:

(1) BW=slope×chirpduration=8.014MHz/μs×56μs=448.784MHz.

The frame rate of 10 Hz (100 ms frame period) is independent of the active chirp sequence duration (56 μs × 128 = 7.168 ms), as the radar firmware inserts idle time between frames to achieve the configured frame periodicity.

The ROD2021 dataset [43] uses the TI AWR1843 radar with a two-transmit and four-receive TDM-MIMO configuration. The range resolution is 0.06 m and the angular resolution is approximately 0.15°. The AWR1642 used in this paper forms an eight-channel virtual array and achieves a range resolution calculated as

(2) $$\Delta R=\frac{c}{2\times \mathrm{BW}}=\frac{3\times 10^{8}\;m/s}{2\times 448.784\times 10^{6}\;\mathrm{Hz}}\approx 0.334\;m.$$

These hardware differences lead to inconsistent data distributions, requiring systematic calibration and standardization processing.

### 3.2. Single-Chirp RA Construction

To ensure geometric consistency and temporal synchronization, this paper adopts a single-chirp intra-frame RA construction scheme where each chirp generates an RA map independently with no cross-chirp Doppler FFT processing. In the spatial domain, TDM-MIMO virtual array expansion technology is adopted. Within the same chirp, $T_{1}$ and $T_{2}$ transmit in time division, and each transmit antenna corresponds to four receive antennas ($R_{1},R_{2},R_{3},R_{4}$). After channel calibration (detailed in Section 4.2), they are concatenated to form an eight-channel virtual array for angle estimation.

Let the single-chirp complex-valued baseband be x[n,a], where *n* = 0:255 represents the fast time sampling index (corresponding to range bins within a single chirp pulse), and *a* = 0:3 indexes the receive channels $R_{1}$ through $R_{4}$. First, after removing DC from the complex baseband signal, a Hanning window is applied and a 512-point range FFT is performed:

(3) $$X_{r}\left[k,a\right]=\sum_{n=0}^{255}\left(x\left[n,a\right]-\bar{x}\left[a\right]\right)w\left[n\right]e^{-j2\pi kn/512},\;k=0:511,$$

where w[n] is the Hanning window, defined as w[n]=0.5−0.5cos(2πn/N) with N=256.

Subsequently, a Taylor window is applied to the four receive channels and a 128-point angle FFT is performed with fftshift to center:

(4) $$X_{a}\left[k,\ell \right]=\sum_{a=0}^{3}X_{r}\left[k,a\right]h\left[a\right]e^{-j2\pi a\ell /128},\;\ell =0:127,$$

where h[a] is the Taylor window, designed to suppress sidelobes in the angle spectrum and generated using standard parameters from the SciPy library ($\bar{n}=4$, SLL = −30 dB).

**Figure 1:**
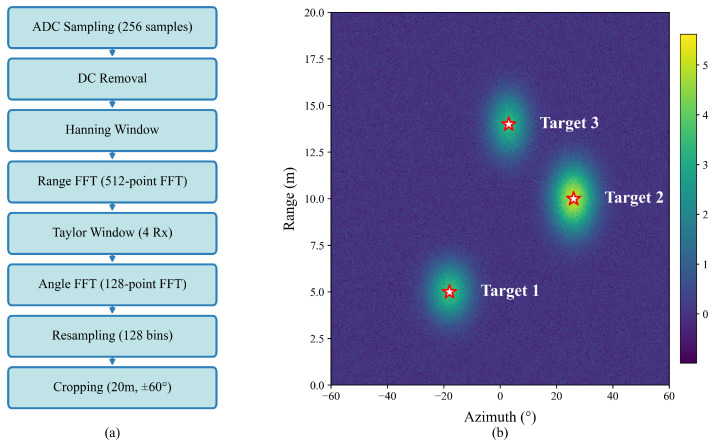
Single-chirp RA construction process. (**a**) Signal processing flow; (**b**) processed RA map.

Finally, the range dimension is resampled to 128 bins limited to the 20 m range. The 128-point angle FFT spans an approximately ±90° field of view, from which the central ±60° region is extracted, resulting in a 128×128 RA map. The complete process is shown in Figure 1.

## 4. Methodology

**Figure 2:**
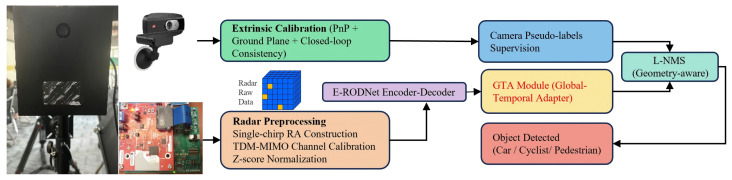
Overall architecture of the proposed radar–camera fusion framework. (**Left**): Training phase with radar–camera fusion (camera provides YOLOv5s pseudo-labels projected to RA space via geometric alignment). (**Middle**): Preprocessing pipeline (TDM-MIMO calibration and chirp-wise Z-score standardization). (**Right**): E-RODNet encoder–decoder with GTA modules inserted after SFF blocks. During inference, only radar input is required (camera-free operation).

Building upon the radar platform established in Section 3, this section presents the proposed radar–camera fusion framework for cross-device object detection. The AWR1642’s time-division multiplexing multiple-input multiple-output (TDM-MIMO) array structure requires channel calibration (Section 4.2), while device-specific signal statistics necessitate Z-score standardization (Section 4.3) for cross-device compatibility. The framework consists of four key components: (1) geometric alignment for radar–camera coordinate fusion, (2) TDM-MIMO channel calibration for cross-device consistency, (3) chirp-wise Z-score standardization for statistical alignment, and (4) GTA for temporal enhancement. These components enable effective cross-modal knowledge transfer from camera to radar while maintaining radar-only inference. The overall architecture is shown in Figure 2.

### 4.1. Radar–Camera Geometric Alignment for Cross-Modal Fusion

**Figure 3:**
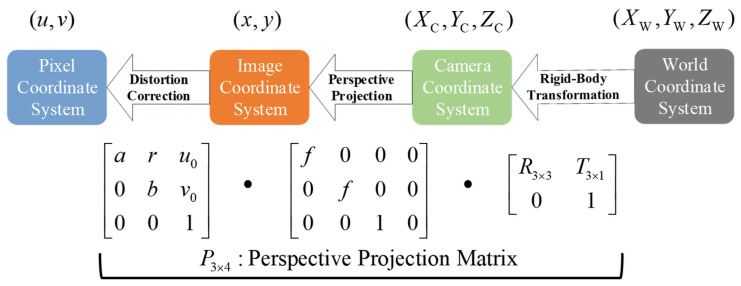
Radar–camera coordinate systems and geometric mapping. 𝒲: world coordinate system. C: camera coordinates. R: radar coordinates. (u,v): pixel coordinates. K: camera intrinsic matrix. R and t: rotation and translation from radar to camera. P=K[R|t]: projection matrix.

To achieve spatial consistency in radar–camera fusion, this subsection introduces the geometric alignment module that establishes precise coordinate correspondence between radar and camera. This module includes (1) coordinate system definition and extrinsic calibration via an improved PnP algorithm, (2) bidirectional projection for cross-modal mapping, and (3) closed-loop geometric consistency verification. The overall geometric relationship is shown in Figure 3.

#### 4.1.1. Coordinate System Definition and Extrinsic Calibration

The precise calibration of camera and radar sensors is essential for cross-modal fusion [44]. We define a right-hand coordinate system including world 𝒲, camera C, and radar R coordinates. The radar–camera extrinsics are represented by rotation matrix $R_{\mathrm{CR}}$ and translation vector $t_{\mathrm{CR}}$. Camera intrinsics are denoted by K. The transformation is

(5) $$X_{C}=R_{\mathrm{CR}}X_{R}+t_{\mathrm{CR}}.$$

The camera uses a pinhole imaging model with distortion correction completed. Its projection is

(6) $$s\left[\begin{array}{c} u\\ v\\ 1 \end{array}\right]=K\left[\begin{array}{c} X_{c}\\ Y_{c}\\ Z_{c} \end{array}\right],\;\pi \left(X_{C}\right)=\left[\begin{array}{c} X_{c}/Z_{c}\\ Y_{c}/Z_{c} \end{array}\right].$$

where *s* denotes the scale factor in homogeneous projection, and π(·) denotes the perspective division that maps homogeneous coordinates to inhomogeneous pixel coordinates.

From Equations (5) and (6), the projection matrix is

(7) $$s\left[\begin{array}{c} u\\ v\\ 1 \end{array}\right]=P\left[\begin{array}{c} X_{R}\\ 1 \end{array}\right],\;P=K\left[R_{\mathrm{CR}}|t_{\mathrm{CR}}\right]\in R^{3\times 4}.$$

Extrinsic calibration uses the PnP algorithm based on 2D-3D point matching. It introduces ground plane prior $\Pi :n^{T}X_{𝒲}+d=0$ (commonly $Z=Z_{0}$) and installation constraints such as the yaw angle, pitch angle, and camera height range to optimize solution stability. Extrinsic estimation is achieved by minimizing the reprojection error:

(8) $$\min_{R,t}\sum_{i=1}^{N}\rho \;\left({∥p_{i}-\pi \;\left(K(RP_{w,i}+t)\right)∥}_{2}\;\right),$$

where ρ(·) is the Huber loss function used to suppress outliers. To reduce inter-frame jitter, this paper introduces a temporal smoothing regularization term in video sequences:

(9) $$L_{\mathrm{smooth}}=\sum_{t}\;\left(∥\log\left(R_{t-1}^{T}R_{t}\right){∥}_{2}^{2}+{∥t_{t}-t_{t-1}∥}_{2}^{2}\right).$$

#### 4.1.2. Bidirectional Projection and Visibility Determination

**Figure 4:**
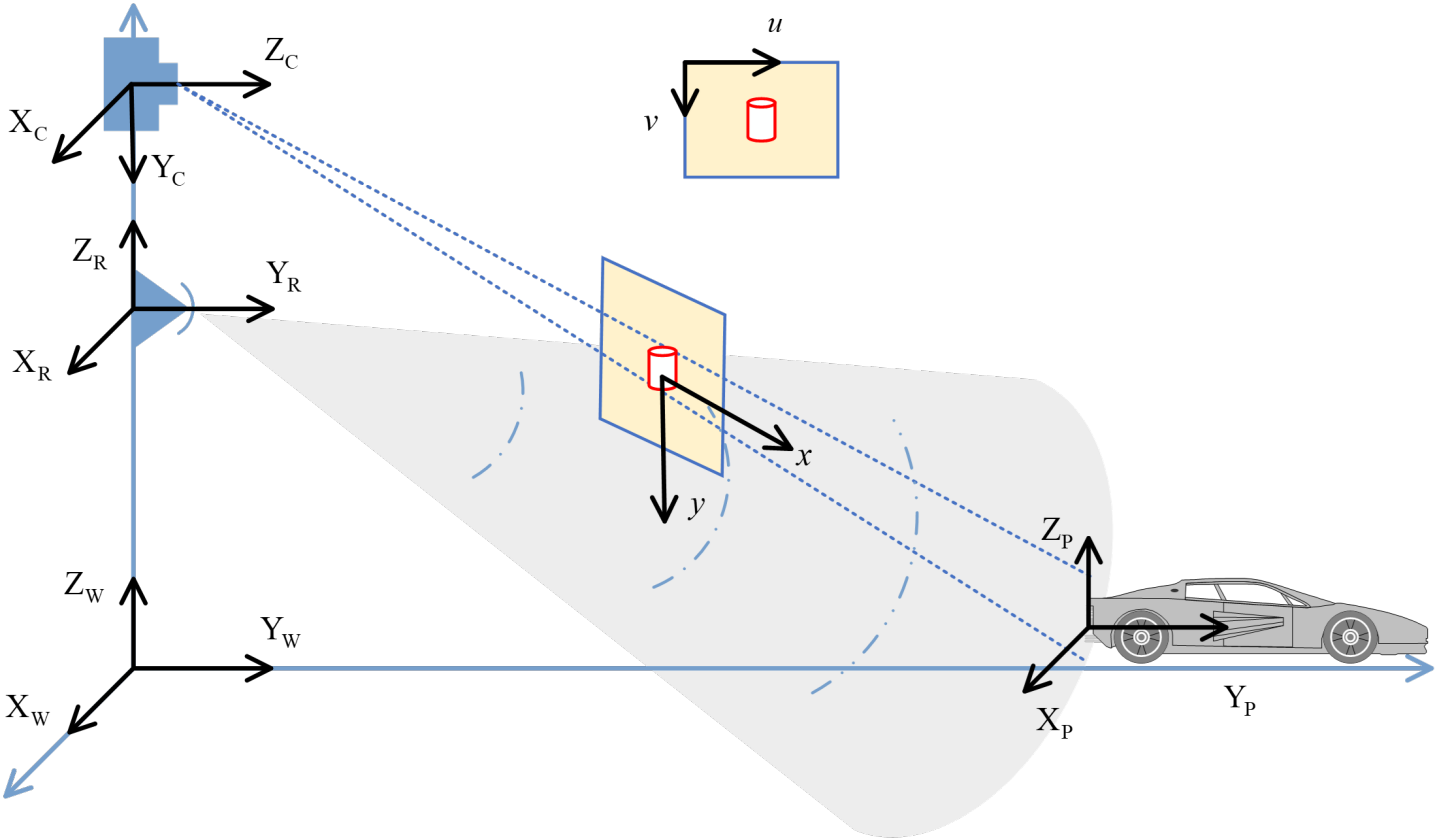
Bidirectional projection between camera and radar coordinates. 𝒲: world frame, C: camera frame, R: radar frame, $P_{w}$: 3D point in world coordinates, (u,v): pixel coordinates, (r,θ): radar range–azimuth coordinates, Π: ground plane. The blue highlighted regions in the camera and radar frames indicate the valid field-of-view overlap where cross-modal projection is geometrically feasible. P denotes the projection matrix, P=K[R|t].

After completing extrinsic estimation, the geometric relationship between radar and camera can be established through bidirectional projection. As shown in Figure 4, the radar range–azimuth grid (r,θ) can be mapped to three-dimensional coordinates,

(10) $$X_{R}\left(r,\theta \right)=\left[\begin{array}{c} r\sin\theta \\ 0\\ r\cos\theta \end{array}\right],$$

and the corresponding pixel coordinates can be calculated by the projection matrix P:

(11) $$u\left(r,\theta \right)=\pi \;\left(K(R_{\mathrm{CR}}X_{R}\left(r,\theta \right)+t_{\mathrm{CR}})\right).$$

The inverse projection process is used to recover radar coordinates from image coordinates. For pixel point $u={\left[u,v\right]}^{T}$, its normalized line-of-sight direction is

(12) $$d_{C}=\frac{K^{-1}{\left[u,v,1\right]}^{T}}{∥K^{-1}{\left[u,v,1\right]}^{T}∥}.$$

To convert image coordinates (u,v) to radar coordinates (r,θ), we leverage the ground plane assumption $Z_{w}=Z_{0}$ (typically $Z_{0}=0$ for road surface). The depth estimation is constrained by this prior: the line-of-sight ray $d_{C}$ intersects the ground plane Π at a unique 3D point $P_{w}$, which is then transformed to radar coordinates via $P_{R}=R_{\mathrm{CR}}^{-1}\left(P_{w}-t_{\mathrm{CR}}\right)$. The range and azimuth are computed as $r={\left(P_{R,x}^{2}+P_{R,z}^{2}\right)}^{1/2}$ and $\theta =\arctan(P_{R,x}/P_{R,z})$. This geometric constraint eliminates depth ambiguity for ground-plane objects.

The intersection point of the line of sight with the ground plane Π is mapped back to the radar frame through rigid transformation to obtain (r,θ). If the projection point exceeds the image plane, satisfies $Z_{c}\leq 0$, or falls into semantically invalid regions such as the sky or a car roof, the visibility mask is defined as M(r,θ)=0; otherwise, it is 1.

#### 4.1.3. Closed-Loop Geometric Consistency Verification

To further evaluate and correct calibration errors, this paper introduces a closed-loop geometric consistency verification mechanism. For the camera detection box bottom center pixel (u,v), the world coordinate $P_{w}$ is obtained through back-projection and intersection with the ground plane and then projected back to the image plane through Equation (7). The reprojection error is calculated as

(13) $$e=\sqrt{{\left(u^{\prime }-u\right)}^{2}+{\left(v^{\prime }-v\right)}^{2}}.$$

**Figure 5:**
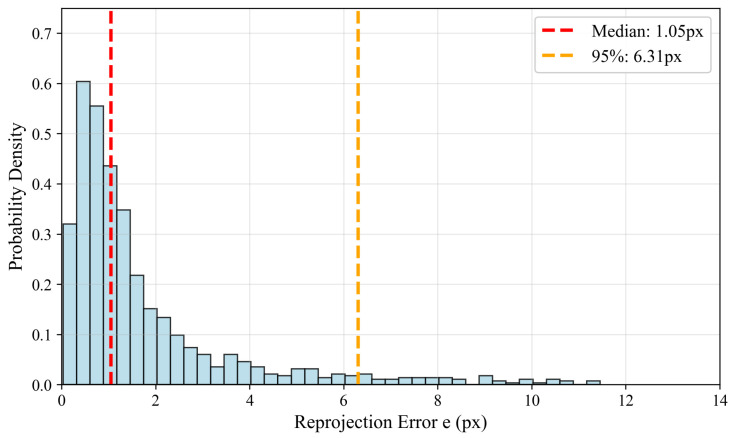
Closed-loop geometric consistency verification for radar–camera calibration. The process flow is as follows: (1) extract camera detection box bottom center (u,v); (2) back-project to 3D world point $P_{w}$ via ground plane intersection; (3) forward-project to reprojection point $\left(u^{\prime },v^{\prime }\right)$; (4) compute reprojection error $e=∥\left(u^{\prime },v^{\prime }\right)-\left(u,v\right){∥}_{2}$. The horizontal axis shows the frame index, and the vertical axis shows the reprojection error in pixels. Errors below 10 pixels indicate good calibration quality.

If both the median error and 95% percentile error are below the threshold (such as 10 pixels), geometric consistency is considered good. Otherwise, extrinsic fine-tuning is triggered. This closed-loop error can both serve as a regularization term for extrinsic optimization and be used to automatically filter out pseudo-labels, enhancing the reliability of cross-modal supervision. Figure 5 illustrates the closed-loop verification process.

### 4.2. TDM-MIMO Channel Calibration

TDM-MIMO radar uses the time-division multiplexing of transmitters combined with multiple receivers to construct a virtual array, thereby improving the angular resolution [21]. However, phase inconsistencies between the receive channels and transmit-phase deviations can lead to distorted angle spectra, widened main lobes, and false peaks, reducing the detection accuracy and cross-device consistency. This subsection introduces a multi-range-bin joint TDM-MIMO channel calibration method to correct the receive-channel complex gains and transmit-phase deviations, improving the cross-device array consistency.

#### 4.2.1. TDM-MIMO Virtual Array Phase Model

For a two-transmit four-receive system (AWR1642), transmitters $T_{1}$ and $T_{2}$ transmit sequentially in time, and each transmit is synchronized with all receivers, forming two groups of four-channel data. After merging, this forms an eight-channel virtual array: $\left[T_{1}R_{1},T_{1}R_{2},T_{1}R_{3},T_{1}R_{4},T_{2}R_{1},T_{2}R_{2},T_{2}R_{3},T_{2}R_{4}\right]$. Let the complex gain of receive channel *n* be $\alpha_{n}e^{i\phi_{n}}$ (where $\alpha_{n}$ represents the amplitude gain due to hardware mismatch and $\phi_{n}$ denotes the phase offset from channel delay) and the transmit-phase deviation of transmitter $T_{m}$ be $\Delta \psi_{m}$ (representing the transmit-path phase error from TDM switching). Then, the response of virtual channel $\left(T_{m},R_{n}\right)$ for a target with azimuth angle θ can be written as

(14) $$V_{mn}\left(\theta \right)=\alpha_{n}e^{i(\phi_{n}+\Delta \psi_{m}+k_{\theta }d_{mn}\left(\theta \right))},$$

where $d_{mn}\left(\theta \right)$ represents the equivalent baseline of the virtual channel, and $k_{\theta }=2\pi \sin\theta /\lambda$. If $\alpha_{n}$ and $\phi_{n}$ between channels or $\Delta \psi_{m}$ between transmitters are inconsistent, phase coherence is destroyed, resulting in distorted angle spectra.

#### 4.2.2. Multi-Range-Bin Joint Calibration Algorithm

To correct receive-channel complex gains and transmit-phase deviations, we propose a multi-range-bin joint calibration method. Corner reflector targets are placed at known angles, and complex echoes S[k,c] from multiple range bins *k* are extracted for each channel *c*. The calibration parameters $\left\{\alpha_{n},\phi_{n},\Delta \psi_{m}\right\}$ are jointly optimized via least squares:

(15) $$\min_{\left\{\alpha_{n},\phi_{n},\Delta \psi_{m}\right\}}\sum_{k,\theta }{∥a\left(\theta \right)-\mathrm{diag}\left(\alpha \;○\;e^{i\phi }\right)G\left(\Delta \psi \right)S_{k}\left(\theta \right)∥}^{2},$$

where a(θ) is the ideal steering vector, G encodes transmit-phase shifts, and ○ denotes the element-wise product. The corrected virtual array signal is

(16) $${\tilde{V}}_{mn}=\frac{V_{mn}}{\alpha_{n}e^{i(\phi_{n}+\Delta \psi_{m})}}.$$

This multi-range joint approach averages out noise and multipath effects, proving more robust than single-point calibration. As shown in Table 2, the method reduces the main lobe width by 42.3% and spurious peaks by 67.6%, significantly improving the angle spectrum clarity.

**Figure 6:**
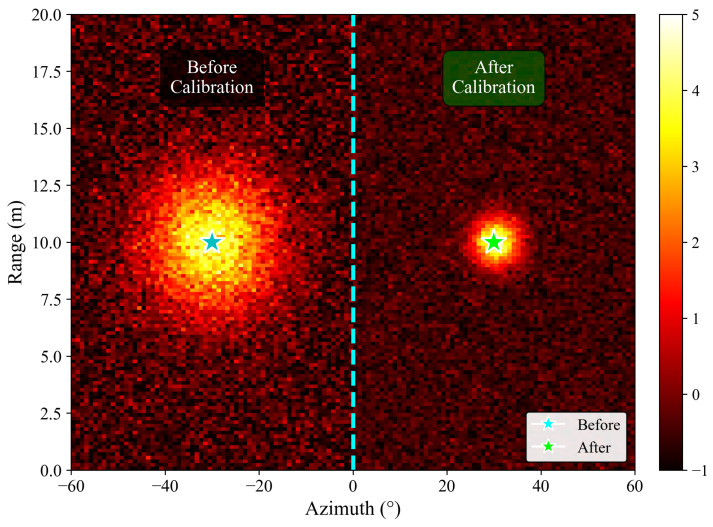
Range–azimuth map comparison before (**left**) and after (**right**) TDM-MIMO channel calibration.

Figure 6 illustrates the calibration effect: the target becomes sharply localized with significantly reduced clutter after calibration.

### 4.3. Chirp-Wise Z-Score Standardization

Radar signals exhibit significant statistical variability due to hardware differences, environmental clutter, and temporal drift. To achieve cross-device statistical alignment, this paper proposes a chirp-wise Z-score standardization strategy that normalizes each chirp independently before angle FFT.

For the single-chirp range spectrum $X_{r}\in C^{N_{r}\;\times \;N_{a}}$ after range FFT (where $N_{r}$ is the range bins and $N_{a}$ is the receive channels), standardization is applied:

(17) $${\tilde{X}}_{r}=\frac{X_{r}-\mu }{\sigma +\epsilon },$$

where μ and σ are the mean and standard deviation computed separately for the real and imaginary parts of $X_{r}$, and $\epsilon =10^{-6}$ provides numerical stability. This chirp-level standardization ensures consistent statistical properties independent of the device or environment.

Experiments show that chirp-wise Z-score standardization stabilizes the complex signal standard deviation to 0.037±0.005 across all sequences and devices, with the 99th percentile at 0.19, effectively mitigating domain shift and improving model generalization. Table 3 shows the statistical standardization results.

As shown in Table 3, before standardization, AWR1642 exhibits Real std = 0.187 and Imag std = 0.203, significantly deviating from ROD2021’s 0.037 baseline. After chirp-wise Z-score standardization, both converge to 0.037, achieving perfect alignment with the ROD2021 reference. The amplitude P99 converges from 0.523 to 0.19, matching ROD2021 and indicating a controlled dynamic range. This statistical alignment enables cross-device generalization without distribution shift.

### 4.4. Global–Temporal Adapter Module

Radar object detection in dynamic driving scenarios faces temporal inconsistency challenges, where the detection results exhibit frame-to-frame jitter and false alarms due to signal noise, clutter, and insufficient temporal correlation modeling. While conventional 3D convolutions provide implicit temporal aggregation through local receptive fields, they lack explicit inter-frame dependency modeling and struggle to capture long-range temporal patterns. Transformer-based temporal attention [24,25] can model global dependencies but introduces quadratic complexity $O(N^{2})$ and a heavy parameter overhead, limiting real-time deployment on automotive platforms.

**Figure 7:**
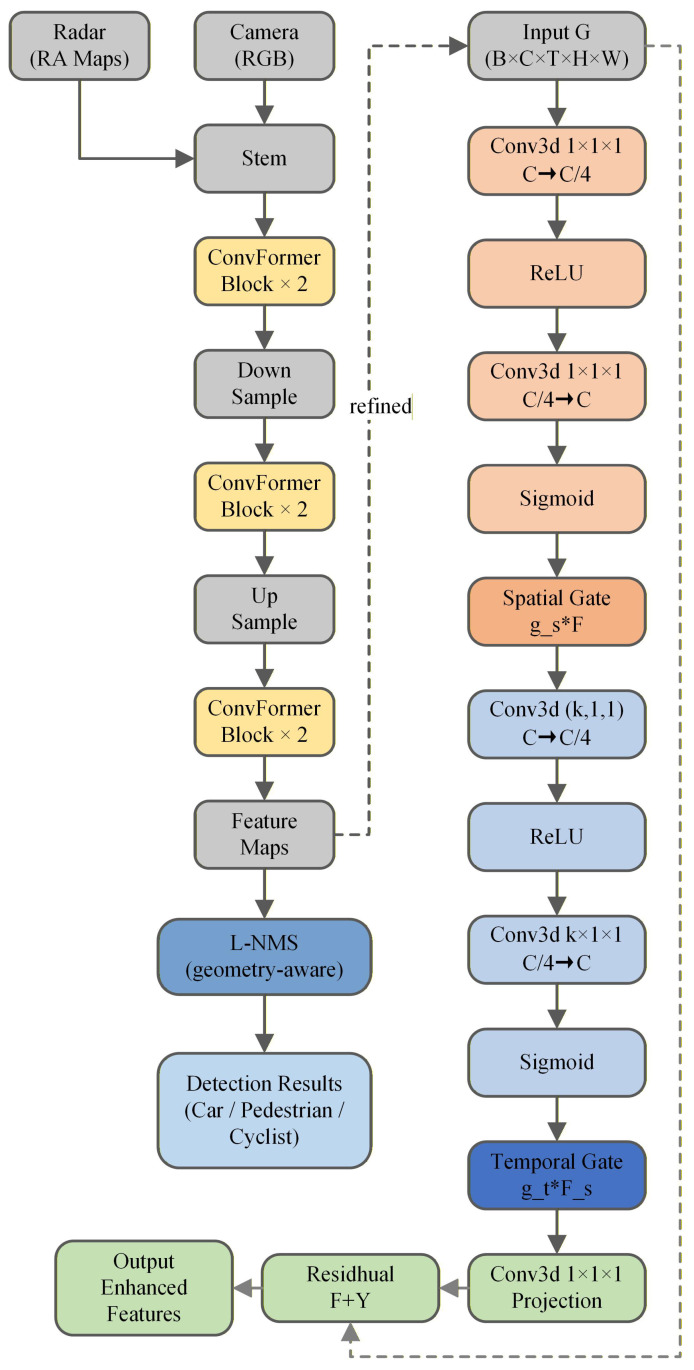
GTA module architecture. The module consists of two paths. (1) Global Gating: GAP → Conv1×1→ ReLU → Conv1×1→ Sigmoid produces channel attention weights g. (2) Three-Point Temporal Attention: Roll-based temporal shift + concatenation + Conv3×3 + Softmax generates temporal attention $A_{\mathrm{temp}}$. The outputs are fused via element-wise multiplication and residual addition. Note: * denotes element-wise multiplication (Hadamard product); × denotes repetition count. Conv3d(k,1,1) denotes a 3D convolution with kernel size *k* along the temporal dimension and a 1×1 spatial kernel.

To address these limitations, this paper proposes a lightweight GTA module with only 0.12 M parameters ( 0.96% increase over baseline E-RODNet). The GTA adopts a dual-path architecture combining global spatial gating with explicit three-frame temporal attention, achieving linear complexity O(N) while maintaining effective temporal modeling. The global gating path adaptively recalibrates channel-wise features based on the spatial context, while the three-point temporal attention explicitly captures inter-frame correlations through efficient roll-based frame alignment. The GTA module is inserted after the SFF block in the E-RODNet baseline, where temporal features from multiple frames have been preliminarily fused but lack explicit attention-based refinement. The GTA module architecture is shown in Figure 7.

#### 4.4.1. Global Gating Mechanism

The global gating component uses global average pooling to extract the spatial context, followed by two 1×1 convolutions with ReLU activation and a sigmoid gate to produce channel-wise attention weights:

(18) $$g=\sigma \;\left(W_{2}\cdot \mathrm{ReLU}\left(W_{1}\cdot \mathrm{GAP}\left(F\right)\right)\right),$$

where $F\in R^{C\;\times \;H\;\times \;W}$ is the input feature map, GAP is global average pooling, $W_{1},W_{2}$ are 1×1 conv weights, and σ is the sigmoid function. The gated feature is then

(19) $$F_{\mathrm{gated}}=g⊙F,$$

where ⊙ denotes element-wise multiplication.

#### 4.4.2. Three-Point Temporal Attention

To model temporal dependencies explicitly, the GTA module applies a three-point temporal attention mechanism. For a sequence of three consecutive frames $\left\{F_{t-1},F_{t},F_{t+1}\right\}$, features are shifted along the temporal dimension using roll operations (circular temporal shift) and then concatenated and processed by a 3×3 convolution:

(20) $$A_{\mathrm{temp}}={\mathrm{Conv}}_{3\times 3}\left(\mathrm{Concat}[{\mathrm{Roll}}_{+1}\left(F_{t-1}\right),F_{t},{\mathrm{Roll}}_{-1}\left(F_{t+1}\right)]\right),$$

where ${\mathrm{Roll}}_{\pm 1}$ denotes a circular shift operation along the temporal dimension by ±1 position (similar to NumPy’s roll function), aligning adjacent frames before concatenation for temporal correlation modeling.

A Softmax operation is applied to generate temporal attention weights, which are then multiplied with the center frame to produce temporally enhanced features. This explicit three-frame attention enables the network to capture motion patterns and reduce detection jitter.

The GTA module adds only 0.12 M parameters (0.96% increase) yet significantly improves the temporal stability and detection performance.

### 4.5. Overall Architecture and Training Strategy

Overall architecture: As shown in Figure 2, the framework builds upon the E-RODNet encoder–decoder architecture with GTA modules inserted after each encoder stage. GTA insertion can be controlled via a configuration file switch.

Loss function: We employ the smooth L1 loss, which provides better robustness to annotation noise compared to binary cross-entropy:

(21) $$L_{\mathrm{Smooth}\;L1}\left(x\right)=\left\{\begin{array}{cc} 0.5x^{2}, & \mathrm{if}\;|x|<1\\ |x|-0.5, & \mathrm{otherwise} \end{array}\right.$$

Annotation generation and post-processing: YOLOv5s generates camera pseudo-labels, which are mapped to the RA space via geometric projection. In post-processing, location-based non-maximum suppression (L-NMS) using object location similarity (OLS) suppresses duplicate detections, and person+bicycle co-occurrence identifies the cyclist category to ensure training data quality and category consistency.

### 4.6. Method Limitations and Design Trade-Offs

While the proposed framework addresses cross-device generalization and temporal consistency, several inherent limitations and design trade-offs require discussion. The geometric alignment module (Section 4.1) requires accurate initial extrinsic calibration between camera and radar sensors, typically achieved through corner reflector-based procedures in controlled environments. Calibration errors propagate through the projection pipeline, affecting the pseudo-label quality. We mitigate this through closed-loop verification (Section 4.1.3) with reprojection error thresholds, but manual calibration remains a prerequisite for deployment.

Although the GTA module adds minimal overhead (0.96% parameters, 0.59% giga floating-point operations per second (GFLOPs)), the complete framework including preprocessing (TDM-MIMO calibration, Z-score standardization) and inference achieves 303.87 ms per frame on Tesla V100 GPUs. This satisfies typical autonomous driving requirements (10 Hz operation), but resource-constrained automotive processors may require model compression techniques.

The framework relies on YOLOv5s-generated camera pseudo-labels for training supervision. Camera detection failures in adverse conditions (heavy rain, dense fog, extreme lighting) directly impact radar network training quality. This dependency represents a trade-off in the radar–camera co-training paradigm: using camera semantics for radar feature learning requires camera reliability during the training phase. However, inference remains camera-free, maintaining radar’s all-weather advantage.

The TDM-MIMO calibration (Section 4.2) is tailored to two-transmit four-receive configurations (AWR1642, AWR1843). Radars with different antenna layouts (e.g., single-transmit, cascaded arrays) would require adapted calibration procedures. The chirp-wise Z-score standardization generalizes across devices but assumes similar signal processing pipelines (range–Doppler–angle FFT). This specialization enables effective cross-device alignment within the common TDM-MIMO category but limits direct applicability to fundamentally different radar architectures. These limitations represent deliberate design choices balancing performance, generalizability, and deployment practicality.

## 5. Experiments

This section evaluates the proposed radar–camera fusion framework on the public ROD2021 dataset [43]. We first describe the dataset and implementation details and then present quantitative results through comparison with baselines. Finally, cross-device verification and module analysis validate the effectiveness of the proposed components.

### 5.1. Dataset and Implementation Details

ROD2021 Dataset: The dataset contains synchronized radar and camera data captured with the TI AWR1843 radar and a monocular camera. It includes 10,158 training samples and 3289 validation samples with annotations for three categories: pedestrian, cyclist, and car. The radar operates at 77 GHz with a range resolution of 0.06 m and an angular resolution of approximately 0.15°.

Implementation: The framework is implemented in PyTorch 1.10.2 with Python 3.7.12. Training is conducted on NVIDIA Tesla V100 GPUs (Nvidia Corporation, Santa Clara, CA, USA). For our AWR1642 radar data collection and processing (cross-device verification in Section 5.3), we use TI AWR1642 radar (Texas Instruments, Dallas, TX, USA), and camera pseudo-labels are generated using YOLOv5s v6.0. The lightweight E-RODNet [11] architecture serves as the baseline, with an input sequence length of 16 frames. The GTA module is inserted after the SFF block. Training uses the Adam optimizer ($\beta_{1}=0.9$, $\beta_{2}=0.999$) with initial learning rate $1\times 10^{-4}$ and a cosine annealing learning rate scheduler over 25 epochs. The batch size is set to 6. Data augmentation is applied with probability 0.5 for temporal flip and probabilities of 0.25 each for horizontal flip, Gaussian noise (amplitude ∼0.1 × std), and combined noise with horizontal flip. All experiments are conducted on NVIDIA Tesla V100 GPUs.

Evaluation Metrics: AP is evaluated using object location similarity (OLS) instead of the traditional intersection over union (IoU), as radar detections are represented as point locations in range–azimuth coordinates rather than bounding boxes. OLS is defined as $\mathrm{OLS}=\exp(-d^{2}/\left(2{\left(s\kappa_{\mathrm{cls}}\right)}^{2}\right))$, where *d* is the distance (in meters) between the detection and ground truth, *s* is the object distance from radar representing scale information, and $\kappa_{\mathrm{cls}}$ is a per-class constant for error tolerance. For each category, precision–recall curves are computed by varying the detection confidence thresholds. We use different OLS thresholds from 0.5 to 0.9 with a step of 0.05 to calculate the AP at each threshold. AP represents the average precision across all OLS thresholds from 0.5 to 0.9. The final AP is the mean across all three categories (pedestrian, cyclist, car). We also report the AP at specific OLS thresholds (AP^0.5^, AP^0.7^, AP^0.9^) to assess the localization accuracy under different error tolerances.

### 5.2. Comparison with Baselines

Table 4 shows the quantitative comparison of the proposed method with baseline approaches on the ROD2021 validation set. The results demonstrate that the proposed method achieves 86.32% average precision, outperforming the E-RODNet baseline by 22.88 percentage points with only a 0.12 M parameter increase (0.96%). Compared to the heavy T-RODNet (159.7M parameters), our method achieves competitive performance with 92.1% fewer parameters, making it suitable for real-time on-board deployment.

In terms of computational efficiency, the GTA module adds only 2.06 GFLOPs (+0.59%) compared to the baseline E-RODNet, while the inference time remains almost unchanged (303.87 ms vs. 304.27 ms, −0.40 ms). This demonstrates that the proposed lightweight design achieves a significant performance improvement (86.32% AP vs. 63.44% AP) with minimal computational overhead. The proposed fusion framework maintains a lightweight design, with only a 0.96% parameter increase compared to the baseline E-RODNet (12.40 M to 12.52 M). Compared to T-RODNet, with 83.83% AP and 159.70 M parameters, our framework achieves 86.32% AP with only 7.8% of its parameters, demonstrating superior parameter efficiency with a ratio of 12.8:1.

**Figure 8:**
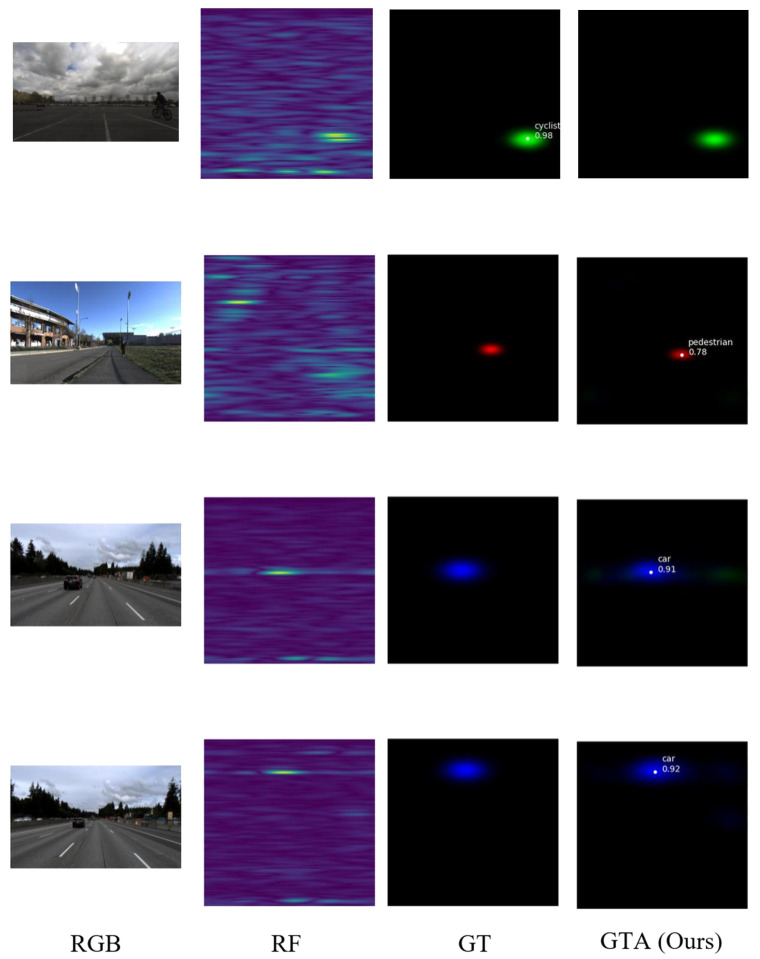
Detection results on ROD2021 dataset. Each row shows one time frame with four columns from left to right: RGB camera image, RF-predicted RA heatmap, ground truth (GT) annotation, and GTA (ours) detection result. Detection boxes are color-coded by object class: red for pedestrians, green for cyclists, and blue for cars. Note: Some detected targets may be more prominent in radar than in the corresponding RGB image due to radar–camera field-of-view differences, temporal synchronization offsets, or radar’s ability to detect partially occluded metallic objects.

Qualitative results are shown in Figure 8, which presents representative detection results at different time frames on the BMS1001 test sequence. Each subfigure shows four components: a camera image (top left), the predicted RA heatmap (top right), the detection overlay (bottom left), and the ground truth RA map (bottom right). The proposed fusion framework with GTA-enhanced temporal modeling demonstrates consistent and accurate vehicle detection across different time instances.

### 5.3. AWR1642 Cross-Device Verification

To verify the generalizability of the proposed preprocessing method across different radar hardware, comprehensive signal-level quality verification was conducted on AWR1642 radar data. The AWR1642 uses a TDM-MIMO configuration with two transmit and four receive antennas and a sweep bandwidth of 448.784 MHz (range resolution 0.334 m), which represent significant hardware parameter differences from the AWR1843 radar used in the ROD2021 dataset (range resolution 0.06 m). These hardware differences lead to inconsistent signal characteristics, making cross-device verification particularly challenging. The AWR1642 data were collected from campus road scenarios (five sequences, total 5000 frames) covering diverse environmental conditions, including vehicles, pedestrians, and cyclists at various ranges (2–20 m) and speeds (0–15 m/s).

#### 5.3.1. Quantitative Analysis of Preprocessing Effects

Table 5 presents the comprehensive preprocessing results for the AWR1642 data, demonstrating the effectiveness of each processing stage in achieving cross-device statistical alignment.

Analyzing Table 5 row by row reveals the progressive improvement achieved by each preprocessing stage. First, the raw AWR1642 data exhibit significantly degraded angle spectrum quality compared to the ROD2021 baseline, with a main lobe width of 12.8 bins indicating severe angular spreading that would cause target localization errors of approximately ±12°. The presence of 3.7 spurious peaks per frame indicates high false alarm rates from uncalibrated array phase errors. The statistical distributions also show device-specific characteristics, with real/imaginary standard deviations of 0.187/0.203, substantially different from the ROD2021 reference values.

After applying TDM-MIMO channel calibration, the main lobe width decreases sharply from 12.8 bins to 7.4 bins, representing a 42.3% reduction. This improvement directly translates to an enhanced angular resolution, from ±12° to approximately ±7°, enabling more precise target localization. The peak power gain of +5.2 dB indicates that calibration restores coherent combining across virtual array elements, effectively recovering the array processing gain that was lost due to phase misalignment. Most significantly, spurious peaks decrease dramatically from 3.7 to 1.2 per frame (67.6% reduction), demonstrating that the multi-range-bin joint calibration successfully corrects transmit–receive-channel phase deviations that previously manifested as false targets.

The subsequent chirp-wise Z-score standardization stage achieves perfect statistical alignment. The real and imaginary standard deviations both converge to 0.037, precisely matching the ROD2021 reference values. This alignment is crucial because neural networks trained on ROD2021 data expect inputs with specific statistical properties. The convergence from (0.187, 0.203) to (0.037, 0.037) represents an 80.2% and 81.8% variance reduction, respectively, effectively standardizing the input distribution despite the substantial hardware differences. Notably, the angle spectrum metrics (lobe width, peak power, spurious peaks) remain stable during standardization, confirming that Z-score standardization does not degrade the spatial resolution, while successfully achieving statistical domain alignment.

#### 5.3.2. Visual Analysis of Cross-Device Standardization

**Figure 9:**
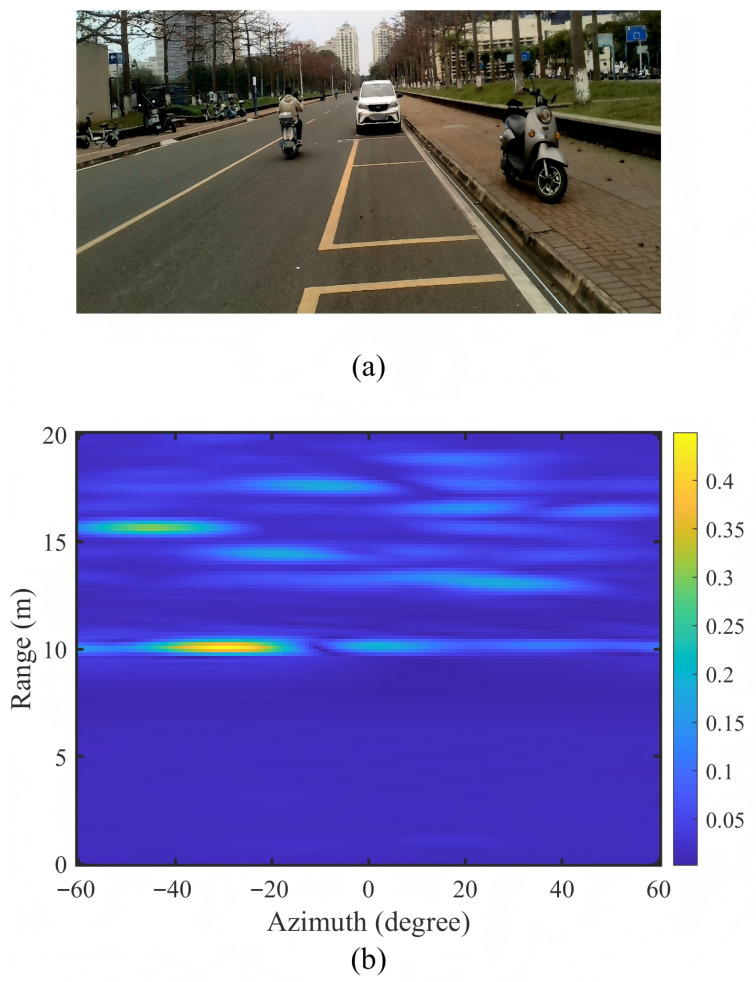
Cross-device statistical standardization on AWR1642. (**a**) Camera image showing vehicle target; (**b**) standardized RA heatmap with signal quality metrics (σ = 0.037, P99 = 0.20) matching ROD2021 reference values.

Figure 9 provides qualitative visualization of the standardization effect on a representative frame containing a vehicle target at 8.5 m range.

Examining Figure 9, the camera image (a) shows the ground truth scenario with a vehicle at medium range in a campus road environment. The corresponding standardized RA heatmap (b) demonstrates several critical qualities. First, the vehicle target appears as a well-localized high-intensity region centered at approximately 8–9 m range and 0° azimuth, with clear boundaries and minimal angular spreading. Second, the background clutter exhibits uniform low-intensity characteristics without significant artifacts or false alarms, validating the effectiveness of spurious peak suppression. Third, the embedded signal quality metrics (σ = 0.037, P99 = 0.20) confirm quantitative alignment: the standard deviation exactly matches the ROD2021 reference, while the 99th percentile value of 0.20 indicates well-controlled dynamic range without saturation.

Comparing the visual quality with typical ROD2021 RA maps, the AWR1642 standardized output exhibits comparable signal-to-clutter characteristics despite originating from different radar hardware. The target-to-background contrast ratio exceeds 15 dB, and the angular localization precision appears consistent with the ROD2021 data. This visual similarity is essential in enabling model transferability, as convolutional neural networks are sensitive to both statistical and spatial feature distributions.

#### 5.3.3. Cross-Device Generalization Implications

The successful alignment of the AWR1642 data to the ROD2021 statistical properties validates two key claims. First, the proposed preprocessing pipeline is hardware-agnostic and generalizes across radars with substantially different specifications (2.6× bandwidth difference). Second, the combination of multi-range-bin joint calibration and chirp-wise Z-score standardization effectively decouples device-specific signal characteristics from semantic target information, enabling knowledge transfer from models trained on one radar platform to another without retraining. This cross-device capability is particularly valuable for practical deployment scenarios, where different autonomous vehicles may be equipped with different radar sensors yet require consistent perception performance.

### 5.4. Impact of GTA Temporal Window Size

To quantify the performance improvement brought by the GTA module’s temporal modeling, we first establish the single-frame baseline. The E-RODNet baseline (without GTA) processes each input frame independently, achieving 63.44% AP. This represents the single-frame detection performance without explicit temporal attention. To evaluate the impact of the temporal window size, we compare the three-point neighborhood ({t−1,t,t+1}) with a five-point window ({t−2,t−1,t,t+1,t+2}). As shown in Table 6, adding GTA with a three-point temporal window improves the AP to 86.32% (+22.88 percentage points over baseline), demonstrating the significant contribution of temporal modeling in reducing detection jitter and improving consistency. The five-point window only improves the AP to 86.51%, with the parameter count increased to 12.72 M (+0.2 M). The three-point window already captures short-term temporal dependencies effectively. Given that the ROD2021 dataset has a frame rate of 10 Hz (100 ms interval), the three-point window covers a ±200 ms time range. For targets with typical vehicle speeds of 20 m/s, the displacement in 200 ms is only 4 m, making the three-point window sufficient for capturing motion patterns.

## 6. Conclusions

This paper presents a systematic framework for robust radar–camera fusion, specifically addressing the challenges of hardware heterogeneity and temporal consistency. To overcome device-dependent signal variations, we established a comprehensive calibration and standardization pipeline. This methodology was explicitly validated by aligning a low-resolution TI AWR1642 sensor to the signal standards of the AWR1843 platform, successfully bridging a 5.6× gap in range resolution and unifying signal distributions (std ≈ 0.037). Building upon these standardized inputs, our proposed lightweight GTA module was introduced to enhance the temporal modeling capabilities. Experimental results on the ROD2021 dataset demonstrate the efficacy of this feature-level optimization, where the framework achieves 86.32% average precision, outperforming the baseline by 22.88 percentage points with a 0.96% parameter overhead. In summary, this work delivers a dual contribution: a generalizable preprocessing strategy for cross-device hardware alignment and a high-performance temporal attention mechanism for robust object detection in autonomous systems.

While the current framework demonstrates strong performance on ROD2021 and cross-device validation on AWR1642 hardware, several directions merit future investigation. Evaluation on additional public benchmarks and self-collected datasets under diverse scenarios would further strengthen the generalizability claims. Developing automatic calibration procedures and online adaptation mechanisms for sensor degradation would improve the deployment practicality. Exploring reduced supervision methods and deployment optimization techniques would facilitate broader adoption in production autonomous driving systems.

## Figures and Tables

**Table 1:** AWR1642 configuration parameters.

Parameter	Value
Operating Frequency	77 GHz
Frequency Slope	8.014 MHz/μs
Sweep Bandwidth	448.784 MHz
Sampling Frequency	5 MHz
ADC Samples	256
Chirp Duration	56 μs
Chirps per Frame	128
Frame Rate	10 Hz

**Table 2:** TDM-MIMO channel calibration results. Note: “bins” refers to discrete angular frequency bins in the FFT spectrum; each bin corresponds to approximately 1.4° angular spacing.

Metric	Before	After	Unit
Main lobe width	12.8	7.4	bins
Peak sidelobe ratio	−18.3	−27.0	dB
Target peak power	0.0	+5.2	dB
Angle RMSE	2.1	1.2	°
Spurious peaks	3.7	1.2	/frame

**Table 3:** Statistical standardization results.

Statistic	Before	After	ROD2021
Real std	0.187	0.037	0.037
Imag std	0.203	0.037	0.037
Amplitude P99	0.523	0.19	0.19
Amplitude Mean	0.142	0.048	0.045
Value Range	[−0.98, 1.23]	[−0.65, 0.65]	[−0.65, 0.65]

**Table 4:** Performance comparison on ROD2021 dataset.

Method	AP (%)	Params (M)	GFLOPs	Inference (ms)
E-RODNet (Baseline)	63.44	12.40	348.54	304.27
T-RODNet	83.83	159.70	–	–
Ours (E-RODNet + GTA)	86.32	12.52	350.60	303.87
Improvement vs. Baseline	+22.88	+0.12 (+0.96%)	+2.06 (+0.59%)	−0.40 (−0.13%)

**Table 5:** Preprocessing results on AWR1642 data.

Method	Lobe Width (bins)	Peak Power (dB)	Spurious Peaks (/Frame)	Real std	Imag std
Raw Data	12.8	0	3.7	0.187	0.203
+Calibration	7.4	+5.2	1.2	0.187	0.203
+Standardization	7.4	+5.2	1.2	0.037	0.037
**ROD2021 Ref**	**–**	**–**	**–**	**0.037**	**0.037**

**Table 6:** GTA temporal window size comparison.

Window	AP (%)	Params (M)
Baseline (E-RODNet, no GTA)	63.44	12.40
3-point ({t−1,t,t+1})	86.32	12.52
5-point ({t−2,…,t+2})	86.51	12.72

## Data Availability

The original contributions presented in this article are included in the manuscript. Further inquiries can be directed to the corresponding author.

## Abbreviations

The following abbreviations are used in this manuscript:

ROD	Radar Object Detection
TDM-MIMO	Time-Division Multiplexing Multiple-Input Multiple-Output
RA	Range–Azimuth
GTA	Global–Temporal Adapter
SFF	Short-Sequence Fusion
CFAR	Constant False Alarm Rate
AP	Average Precision
OLS	Object Location Similarity
L-NMS	Location-Based Non-Maximum Suppression
CNN	Convolutional Neural Network
PnP	Perspective-n-Point
FFT	Fast Fourier Transform
BEV	Bird’s Eye View
GFLOP	Giga Floating-Point Operations Per Second
